# Cold Hardiness in Trees: A Mini-Review

**DOI:** 10.3389/fpls.2018.01394

**Published:** 2018-09-20

**Authors:** Michael Wisniewski, Annette Nassuth, Rajeev Arora

**Affiliations:** ^1^United States Department of Agriculture – Agricultural Research Service, Kearneysville, WV, United States; ^2^Department of Molecular and Cellular Biology, University of Guelph, Ontario, ON, Canada; ^3^Department of Horticulture, Iowa State University, Ames, IA, United States

**Keywords:** freezing tolerance, ice nucleation, cold acclimation, deacclimation, dormancy, C-repeat binding factor (CBF), *DAM* genes, antifreeze protein (AFP)

## Abstract

Significant advances have been made in our understanding of the regulation of cold hardiness. The existence of numerous biophysical and biochemical adaptive mechanisms in perennial woody plants and the complexity their regulation has made the development of methods for managing and improving cold hardiness in perennial woody plants has been very difficult. This may be partially attributed to viewing cold hardiness as a single dimensional response, rather than as a complex phenomenon, involving different mechanisms (avoidance and tolerance), different stages (mid-winter vs. late winter), and having an intimate overlap with the genetic regulation of dormancy. In particular separating the molecular regulation of cold hardiness from growth processes has been challenging. ICE and C-repeat binding factor (CBF), transcription factors (Inducer of CBF expression and CRT-binding factor) have been shown to be an important aspect in the regulation of cold-induced gene expression. Evidence has emerged, however, that they are also intimately involved in the regulation of growth, flowering, dormancy, and stomatal development. This evidence includes the presence of CBF binding motifs in genes regulating these processes, or through cross-talk between the pathways that regulate them. Recent changes in climate that have resulted in erratic episodes of unseasonal warming followed by more seasonal patterns of low temperatures has also highlighted the need to better understand the genetic and molecular regulation of deacclimation, a topic of research that is only more recently being addressed. Environmentally-induced epigenetic regulation of stress responses and seasonal processes such as cold acclimation, deacclimation, and dormancy have been documented but are still poorly understood. Advances in the ability to efficiently generate large DNA and RNA datasets and genetic transformation technologies have greatly increased our ability to explore the regulation of gene expression and explore genetic diversity. Greater knowledge of the interplay between epigenetic and genetic regulation of cold hardiness, along with the application of advanced genetic analyses, such as genome-wide-association-studies (GWAS), are needed to develop strategies for addressing the complex processes associated with cold hardiness in woody plants. A cautionary note is also indicated regarding the time-scale needed to examine and interpret plant response to freezing temperatures if progress is to be made in developing effective approaches for manipulating and improving cold hardiness.

## Introduction

Ever since the first microscopic observations of the freezing response of cells were made in the latter part of the 19th century and early 20th century (Molisch, 1897; Wiegand, 1906), and it was discovered that plant cells undergo cytorrhysis rather than plasmolysis in response to freezing, an elusive search has been conducted to develop a complete and integrated understanding of cold hardiness and freezing tolerance in plants (Wisniewski et al., 2003; Gusta and Wisniewski, 2013; Arora, 2018). Despite thousands of reports and countless reviews, reliable approaches to improving freezing tolerance, without affecting other aspects of plant development, have yet to be developed, either at the molecular/genetic level or the physiological level. New technologies have allowed us to understand plant response to low temperatures in greater and greater detail, but the picture has greatly increased in complexity.

The lack of progress may be partially attributable to two factors. One factor is interpreting cold hardiness as a singular on/off response rather than a combination of many diverse mechanisms that involve significant structural, biochemical, and genetic adjustments, as well as the complexity of manipulating cold hardiness without having a negative impact on other plant developmental processes. The characteristics of these components are species-specific (often genotype-specific), potentially under separate genetic control. Therefore, it is essential when investigating plant cold hardiness to be cognizant of what aspect of the process is being studied and its potential impact on the aspect of cold hardiness that is deemed to be critical for survival. The second factor, is related to the difficulty of studying the biology of organisms at low temperatures, where the kinetics of reactions, and the time required for processes to reach an equilibrium can be problematic when conducting experiments. As noted in Gusta and Wisniewski (2013), the admonition made by Felix Franks in his book on the biophysics of water at low temperature (Franks, 1985), is very relevant. “Too frequently experimental observations on highly complex systems are based on measurements performed under non-equilibrium conditions and rationalized in terms of elementary textbook science. The degree of undercooling (mostly presented using the incorrect terminology, supercooling), the mechanism of ice nucleation, the growth and type of crystals, their size and distribution, the flow properties of the unfrozen matrix, and long-term effects of aging, all need to be taken into account.” The book published by Franks, 1985 still serves as an invaluable primer on the low temperature biology.

Cold hardiness adaptations in plants have been divided into two general categories, tolerance and avoidance. The former involves transcriptomic reprogramming and a host of subsequent biochemical changes that allow plants to tolerate freezing temperatures and the presence of ice in their tissues, while the latter involves mechanisms that allows pockets of water to remain undercooled (deep supercooling) to very low, sub-zero temperatures (-20 to -40°C), so that the supercooled cells are not exposed to the dehydrative effects associated with a freeze tolerance response (often referred to as extracellular freezing). Deep supercooling is characteristic of the dormant buds of many woody perennials and the xylem parenchyma cells of many temperate tree species. The terms freeze tolerance and avoidance, however, are somewhat inaccurate, though widely used, as in both cases cells are avoiding freezing. In the case of freezing tolerance, this is accomplished by the loss of cellular water to extracellular ice, which then decreases the freezing point of the cytoplasm. In the second case, water is not relocated to sites of extracellular ice, even though extracellular ice is present, but instead remains in a metastable condition, and prone to “flash” intracellular freezing (Fujikawa et al., 2009; Wisniewski et al., 2014b). Processes relevant to these strategies are ice nucleation and propagation (Wisniewski et al., 2009, 2014b), the ability to specifically determine where ice crystals are initiated in plant tissues and what shape they form as they grow (McCully et al., 2004), and the formation of cryoprotective and antifreeze compounds (Duman and Wisniewski, 2014).

Despite the complexity of plant cold hardiness, considerable progress has been made in understanding the various components that comprise cold hardiness (Gusta and Wisniewski, 2013). This mini-review highlights one area where considerable progress has been made in understanding the genetic regulation of cold acclimation, and another topic, deacclimation, that is deserving of considerable more focus due to the erratic patterns of warming and cooling temperatures that have developed in the context of climate change. These highly variable weather patterns have had a major impact on dormancy, cold acclimation, and chilling requirements.

## The Molecular Regulation of Plant Cold Hardiness

**FIGURE 1:**
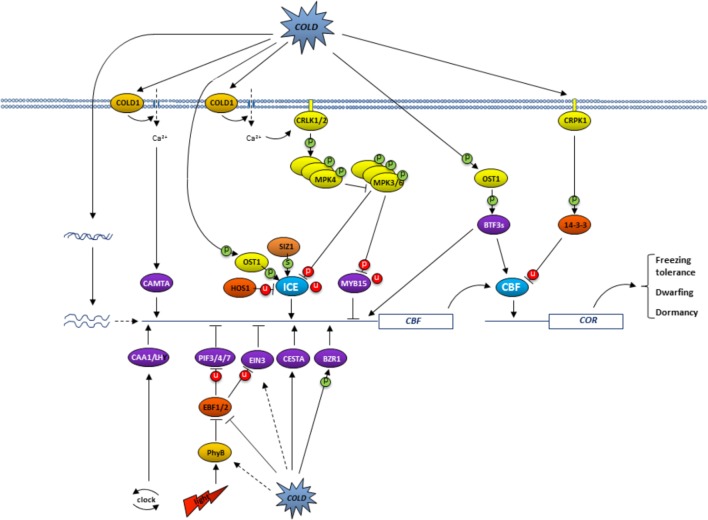
Overview of the regulation of the CBF pathway in *Arabidopsis*. Low temperatures trigger plasma membrane rigidification which leads, presumably via COLD1-like protein, to the opening of Ca^2+^ channels. The resulting higher calcium levels activate CRLK1/2 (calcium/calmodulin-regulated receptor-like kinase; Yang et al., 2010a,b). In turn, this CRKL1/2 triggers the MEKK1-MMK2-MPK4 cascade to ultimately increase ICE activity, because it inhibits the phosphorylation of ICE1 by MPK3/6 and subsequent ICE1 ubiquitination and degradation (Li et al., 2017a; Zhao et al., 2017). ICE activity is further regulated by low temperatures via OST1 (open stomata 1), induced phosphorylation (Ding et al., 2015), and SIZ1-induced sumoylation (Miura et al., 2007), both of which interfere with HOS1 (high expression of osmotically responsive protein 1), directed ubiquitination and subsequent degradation of ICE (Dong et al., 2006). The resulting active ICE directs *CBF* expression. Low temperature activated phosphorylation of 14-3-3 proteins by CRPK1 cause the degradation of CBF proteins (Liu et al., 2017). In contrast, cold-induced OST1-directed phosphorylation of BTF3s promotes its binding to CBFs and thereby prevents CBF degradation (Ding et al., 2018). Photoperiod regulates *CBF* expression via red light perception by PhyB and subsequent degradation of PIF3 (phytochrome-interacting factor 3), thereby relieving its inhibition of *CBF* expression (Jiang et al., 2017), whereas the circadian clock regulates CCA1 and LHY activity (Dong et al., 2011). Interestingly, PIF3 stability is increased by low temperatures, presumably at a later time to downregulate *CBF* expression (Jiang et al., 2017). PIF4/7 and EIN3 (Ethylene insensitive 3) downregulate whereas BZR and CESTA upregulate *CBF* expression, but how this is triggered is not yet known (Shi et al., 2018). Phosphorylation, sumoylation and ubiquitination events are indicated by P, S and U, respectively, with activating modifications in green and inhibiting modifications in red.

Plants cannot move but rather must adapt to a stressful environment. Genes encoding transcription factors in the model plant *Arabidopsis* constitute 6–10% of their genome, compared to 5% in humans, and it is therefore not surprising that adaptation to stresses in plants includes a dramatic change in transcriptional cascades (Pireyre and Burow, 2015). The C-repeat binding factor (CBF), transcription factor pathway has been demonstrated to play an exceptionally important role in plant cold acclimation, a process in which low temperatures lead to biochemical and physiological changes that confer freezing tolerance. These changes are largely associated with the expression of so-called Cold Responsive (COR) genes. In *Arabidopsis*, two or three CBFs co-regulate, often with other transcription factors, more than two-thirds of *COR* genes (Shi et al., 2017). The increase in frost tolerance under ambient conditions that has been demonstrated to occur in many plants as a result of *CBF* overexpression, and the decrease in frost tolerance in *CBF* triple mutants (Jia et al., 2016; Zhao et al., 2016), further underscores the importance of *CBF* genes. *CBF* overexpression in herbaceous and tree species, however, can also reduce growth and induce dormancy (Wisniewski et al., 2011, 2014a), thus it is not surprising that CBF activity is tightly regulated and exhibits only short periods of elevated presence in an active form. This regulation occurs at various levels, including transcriptional (transcript quantity and variant), translational (protein quantity), and post-translational (protein activity), and can have an immediate effect, because many changes are made to pre-existing molecules. Detailed insights into the regulation of the CBF pathway in *Arabidopsis* has only recently been emerging (**Figure 1**), while limited information for other plant species suggests they have similar but unique regulatory processes of their own. An overview of the information on *Arabidopsis*, and woody plants when available, is presented in the current mini-review.

Low temperature-induced chromatin modification provides physical access to certain genes and allows their transcription. This apparently includes access to and activation of *COR* genes by CBFs (Park et al., 2018). *CBF* gene expression itself is regulated by a large number of transcription factors (Shi et al., 2018). Transcriptional activators include ICE1 and 2 (Inducer of CBF expression 1 and 2), CAMTA 3 and 5 (Calmodulin binding transcription activator 3 and 5), CESTA, BZR1 (Brassinazole-resistant 1), and CCA1/LHY1 (circadian clock-associated 1/late elongated hypocotyl). In contrast, CBF transcription repressors include MYB15, EIN3 (Ethylene insensitive 3), PIF3 (phytochrome-interacting factor 3), and PIF4/7. The activity of these transcription factors is modulated by low temperature, light, and/or a circadian clock in such a way that it results in *CBF* expression only at specific times during constant low-temperature conditions (**Figure 1**). Each transduction most likely also involves one or more hormones (Eremina et al., 2016; Barrero-Gill and Salinas, 2017; Li et al., 2017b; Zhou et al., 2017), whereby CBFs also affect hormone levels (Li et al., 2017c), but details are currently relatively sparse. The fact that a gradual or rapid decrease in temperature has slightly different effects make the CBF pathway even more complicated (Kidokoro et al., 2017).

In addition to transcript levels, the type of transcripts can also be altered in response to a cold period. Approximately 60% of intron-containing genes in *Arabidopsis* were reported to undergo alternative splicing (Marquez et al., 2012), especially under stress conditions. Recent RNAseq analysis for *Arabidopsis* identified the often rapid cold induction of alternative splicing (AS) of over 2,400 genes, with over 1,600 regulated only at the AS level and therefore not detected in most previous analyses (Calixto et al., 2018). *CBF* genes do not have introns, thus AS does not directly affect them, however, alternatively spliced transcripts have been detected for PIF7, PHYB, and CAMTA3 (Calixto et al., 2018), which may alter *CBF* expression.

Once produced, the stability of CBF1/3 proteins is downregulated by their interaction with cold-induced CRPK1-phosphorylated 14-3-3 protein (Liu et al., 2017), and upregulated by their interaction with cold-induced OST1-phosphorylated basic transcription factor 3/BTF3-like protein (BTF3/BTF3L; Ding et al., 2018). While some reports have suggested that *Arabidopsis* CBF1-3 are equally important, others suggest that *Arabidopsis* CBF2 and CBF3 play a more important role in directing the cold response (Jia et al., 2016; Zhao et al., 2016; Shi et al., 2017), and adaptation to low temperature in natural populations (Gehan et al., 2015). This apparently occurs through the employment of different regulons. Whereas the main genes upregulated in CBF2 overexpressing plants were related to lipid localization, starch metabolic process, light stimulus response, and regulation of transcription, the genes regulated by CBF3 were mainly related to oxidative stress response (Li et al., 2017c).

The presence of a similar pathway in perennial woody plants, such as poplar, apple, grape, *Prunus* sp., and eucalyptus, is suggested by the identification of usually a larger number of *ICE*- and *CBF*-like genes, increasing the possible further delineation of functions (Wisniewski et al., 2014a). Investigations into their regulation found that *ICE* RNA levels are not much affected by treatments, suggesting an emphasis on regulation of *ICE* by post-translational modifications (Wisniewski et al., 2014a). *CBF* expression is often induced by low temperatures and/or drought or high salt (Wisniewski et al., 2014a), can be affected by the circadian clock (Artlip et al., 2013), and, for some *CBFs*, is induced by a continuous cold treatment later and/or for a longer time period than reported for the *Arabidopsis CBFs* (Xiao et al., 2006, 2008; Artlip et al., 2013; Leyva-Pérez et al., 2015; Li et al., 2017d). AS was determined to be a prevalent occurence in the transcription of many genes in apple, orange, and grape, with genes in grape plants showing the most AS events (Sablok et al., 2017). Not much is currently known about AS events, however, in *ICE*, or *COR* genes in low temperature conditions, except for *ICE* transcripts in grape (Rahman et al., 2014). AS, however, has been suggested to regulate responses to environmental stresses in many plants, including Western poplar (*Populus trichocarpa*) (Filichkin et al., 2018). Amino acid motifs thought to be involved in post-translational modifications have been identified in predicted sequences for ICE and CBF proteins, and functional studies suggest that they are important (Feng et al., 2012; Nguyen et al., 2016; Carlow et al., 2017), but much more studies are needed to determine when and how they regulate ICE and CBF activity. Together the collective studies suggest that woody perennial plants have a CBF-like pathway similar to *Arabidopsis* (Benedict et al., 2006), even including a trade-off between growth and cold stress tolerance (Ibanez et al., 2010; Tillett et al., 2011; Nguyen et al., 2016) but details of *CBF* regulation in woody plants is still very limited.

## Prospects for the Generation of Plants with Enhanced Freezing Tolerance

The tight regulation of CBF activity is largely lost in transgenic plants using a *CBF* construct driven by a constitutive 35S promoter, thus the use of natural promoters is preferred. Recent reports suggest that plants with constitutive brassinosteroid (BR) response display higher *CBF* expression but no signs of growth retardation (Li et al., 2017b). Therefore, it may be possible to avoid the reduced growth associated with *CBF* overexpression (Artlip et al., 2014), if the “correct” CBF or CBF regulators are utilized. Recently, Dong et al. (2017) conducted a meta-analysis of the effect of *CBF* overexpression on temperature stress tolerance and related responses. In that study data from 75 published articles were analyzed to determine the impact of a host of factors such as origin of the *CBF* gene, promoter used to drive expression, the method of stress evaluation, etc., on temperature response and associated indicators, such as electrolyte leakage, growth, chlorophyll fluorescence, sugar and proline levels, etc., Results indicated that 7 of 8 measured variables were significantly modulated in *CBF* (*DREB*)-transgenic plants, while two of the eight parameters were only modulated in non-stressed plants. The measured parameters were modulated by 32% or more by various experimental variables. The modulating variables included, acclimated vs. non-acclimated, type of promoter, duration of stress and its severity, source of the donor gene, and whether the donor and recipient were the same genus. *CBF* overexpression had a consistent negative impact on plant height, a reduction in electrolyte leakage, and positive impact on survival. The impact was evident in both acclimated and non-acclimated plants, although the greatest impact was observed in acclimated plants. Such analyses may provide a more comprehensive understanding of how to best utilize *CBF* genes with modified promoters to improve freezing stress tolerance.

An alternative, enterprising approach is the modification of endogenous *CBF* genes into variants that lead to a higher frost tolerance using a clustered regularly interspaced short palindromic repeat (CRISPR)/CRISPR-associated protein 9 nuclease (Cas9)-like system which has recently been optimized for use in vegetatively propagated perennial plants (Chen et al., 2018). For example, existing sequences could be modified based on variants present in more frost tolerant cultivars or species (Carlow et al., 2017; Li et al., 2017d), and thereby change their regulation. Because of the precise change, the resulting plants may not be considered genetically modified by government regulatory agencies and may be more acceptable to the general public.

## Deacclimation (DA) Response, a Critical Factor for Winter-Survival

The ability to increase freeze-tolerance in temperate- and boreal-zone woody perennials (fruit and forest tree species) via autumnal cold acclimation is undoubtedly the first line of defense against harsh and long winters. Seasonally induced freeze-tolerance is lost under relatively warmer conditions in a process called ‘deacclimation’, a process that typically occurs in response to spring-warming. The maintenance of a sufficient level of cold-induced freeze-tolerance until the danger of killing frosts is passed, however, is an imperative to avoid frost-damage. For example, erratic temperature fluctuations, i.e., sudden winter-warming or premature spring-like conditions followed by more “normal” freezing temperatures, could render partially or fully deacclimated tissues vulnerable to freeze-damage. Indeed, the frequency of such fluctuations has been increasing (Jentsch et al., 2007; IPCC, 2014), and some of the most devastating killer-frosts across North America have been attributed to such events, e.g., Easter freeze of 2007 (Gu et al., 2008), Mother’s Day freeze of 2010, killer frost of 2012, and the polar vortex of 2014. Field simulations of winter-warming events have also confirmed their damaging effects on overwintering perennials (Taulavuori et al., 1997; Bokhorst et al., 2009, 2010).

## Dormancy Status and Spring Phenology (Budbreak) in Relation to DA

Temperate trees have evolved the ability to tolerate harsh winters by undergoing a period of endodormancy (rest), during which cold-acclimated meristems are less prone to DA when trees are exposed to unseasonal episodes of warming (Kalberer et al., 2006, 2007a,b). Buds of native temperate and boreal trees must satisfy a genetically defined chilling requirement to exit endodormancy (Richardson et al., 1974). Post-endodormancy, buds enter an ecodormant state where they must be exposed to a genetically defined threshold of warming (‘heat units’) (Charrier et al., 2011), for the resumption of meristematic activity and growth to occur (budbreak or spring phenology). Ecodormant buds are substantially more sensitive to warmer temperatures and DA than endodormant buds (Kalberer et al., 2007a). This sensitivity increases progressively as the period of ecodormancy increases, finally culminating in complete DA and spring budbreak (Kalberer et al., 2006; Arora and Taulavuori, 2016). Any shift in this annual cycle of spring phenology could potentially increase the risk of trees encountering frost injury (Vitasse et al., 2014 and references therein). Whether an increased risk occurs depends on a variety of internal factors, including species, chilling requirement, the genetic ability to resist deacclimation in response to transient, unseasonal episodes of warm temperatures, and the capacity to reacclimate. External (environmental) mitigating factors include, temperature fluctuations (intensity and timing), and the region/site (latitude and altitude) where the trees are located (Pagter and Arora, 2013; Arora and Taulavuori, 2016; Vitasse et al., 2018b). The sensitivity of ecodormant buds to deacclimating temperatures has also been reported to increase with increasing photoperiod in spring in species such as European beech (*Fagus sylvatica*) (Vitasse and Basler, 2013). There is ample evidence that warming trends in recent decades have advanced spring budbreak and leaf development in many plant species growing in cold regions (Penuelas and Filella, 2001; Menzel et al., 2006, and references therein). Some studies have indicated, however, that the degree of advancement in spring phenology appears to be declining in the recent years (Yu et al., 2010; Fu et al., 2015).

Advances in spring phenology due to climate change can occur under two scenarios. In the first scenario, a faster than normal accumulation of heat-units by ecodormant buds occurs due to earlier and warmer spring-like temperatures (Cleland et al., 2007). This could render prematurely deacclimated buds vulnerable to subsequent spring frosts. The second, somewhat ignored and paradoxical scenario, involves a more rapid fulfillment of chilling requirement due to warmer winter temperatures than has occurred in more typical, historical winters in certain regions. For example, tree species in northern latitudes or high elevations could experience a greater level of ‘dormancy-breaking chill units’ since the warmer winter temperatures could expose trees to temperatures >0°C that are more effective in breaking endodormancy and reduce exposure to sub-freezing temperatures that do not contribute to chill unit accumulation (Hanninen, 2006). Based on this premise, spring phenology could be expected to advance more rapidly in historically colder areas under warming climate conditions. This would result in premature deacclimation and a greater risk of spring freeze-damage. Indeed, Vitasse et al. (2018a) reported that spring phenology in fruit (apple, cherry), and forest (Norway spruce and European beech) trees has advanced at a faster rate during 1975–2016 at fifty high elevation locations in Switzerland than in other temperate locations. The authors argued that even if the frequency and severity of late spring frosts remains unchanged in the future or changes less than the spring phenology of plants, deacclimated organs may be more exposed to a greater number of freeze-damage events (Vitasse et al., 2018a).

On the other hand, proponents of a possible decline in the advancement or delay in spring phenology by warming climate make their case as follows. They suggest that elevated winter temperature may result in a chilling-deficit, i.e., reduced duration and/or sum of cold. And since heat unit requirement for spring phenology is believed to be inversely correlated with the chill accumulation during dormancy (Harrington et al., 2010; Laube et al., 2014), any reduction in accumulated chilling would result in higher heat-unit requirement, thus slowing down the advance in, or delaying, the spring leaf-unfolding (Yu et al., 2010; Fu et al., 2015). One of these studies (Fu et al., 2015) noted that while spring phenology for seven deciduous forest tree species had advanced by ∼4 d during 1980–1994 in Europe, this response has decreased by ∼40% (to 2.3 d) during 1999–2013 (Pan European Phenology Network). A caveat that must be added for such an observation to be practically and widely applicable, however, is that the temperatures during warmer winters have to be high enough to cause real chilling deficit, i.e., either negate accumulated chilling or be ineffective to meet the chilling requirement.

Although the focus of this mini-review is not on bud dormancy, it is relevant to note that several studies have associated the genetic regulation of chilling requirement and dormancy with *Dormancy Associated MADs-box* (*DAM*) genes in peach (Bielenberg et al., 2008; Li et al., 2009) apple (Wu et al., 2017a), pear (Saito et al., 2013), apricot (Sasaki et al., 2011), leafy spurge (*Euphorbia esula*) (Horvath et al., 2010), kiwifruit (Wu et al., 2011, 2017b), and tea plant (*Camellia sinensis*) (Hao et al., 2017). Bud-dormancy associated candidate genes have also been identified in blackcurrant (*Ribes nigrum*) by Hedley et al. (2010) and Yordanov et al. (2014) identified an *early budbreak* (*EBB*) gene in poplar that was an APETALA2/Ethylene responsive transcription factor responsible for early bud flush.

C-repeat binding factor-binding motifs have been identified in promoters of several *DAM* genes and an *EBB* homolog in apple (Wisniewski et al., 2015a), as well as other plant species. A comprehensive analysis of *DAM* genes in the ornamental woody plant, *P. mume* (Chinese plum, Japanese apricot) demonstrated an interaction between *CBF*s and *DAM* genes, especially *PMCBF1 - PMDAM1* (Zhao et al., 2018) and *CBF* expression lowered whereas MADS-box gene (1 and 3) expression increased in almond flower buds after bud break (Barros et al., 2012). Wisniewski et al. (2015a) noted that overexpression of a peach *CBF* gene (*PpCBF1*) in apple altered the expression of *DAM*, *EBB*, and *RGL* (DELLA) genes and that some members of each of these gene families contained C-repeat regions in their promoter regions that are the target sites for CBF. They provided a model linking *CBF* expression with the regulation of dormancy, bud-break, freezing tolerance, and growth. Interestingly, a subsequent study indicated that the impact of the apple transgenic rootstock overexpressing *CBF* was not graft-transmissible and thus did not affect the cold hardiness of dormancy of the scion cultivar grafted to the transgenic ‘M.26’ rootstock, although growth and flowering were significantly impacted (Artlip et al., 2016).

Current research has highlighted the impact of dormancy status and spring phenology on the propensity of trees to deacclimate. Spring-phenology is an outcome influenced by both the chilling and heat unit requirements of overwintering tree species. The fact that chill- and heat-units can be satisfied by the same temperatures for certain species (Cooke et al., 2012 and references therein) makes their combined effect on spring phenology even more complex. It is therefore critical to include dormancy-status and the interactions between chilling and heat requirements as key parameters in models designed to predict the relationship between deacclimation response and freezing-tolerance.

## Future Directions

The past 50 years of research has provided a wealth of information on the genetic and molecular regulation of plant cold hardiness, as well as the regulation of dormancy. These advances have been fueled by new technologies associated with high-throughput sequencing, genetic mapping, and transformation technologies. In particular the regulatory role of *CBF* genes in freezing tolerance, and of *DAM* genes in the regulation of chilling requirement stand out as major advances. The discovery that CBF activity is regulated continually and at various levels, helps explain, at least in part, why slightly different treatment of plants with respect to light, duration and rate of low temperature treatment for example, lead to different outcomes with respect to their frost tolerance. Notable advances have also been made with the use of high-resolution infrared thermography in our understanding of ice nucleation and propagation (Wisniewski et al., 2014b, 2015b), and the properties of antifreeze proteins (Duman and Wisniewski, 2014). Despite these advances, significant improvements in plant cold hardiness have been elusive and problematic due to the complexity of this trait and its intimate connection to other plant developmental processes, especially growth and flowering. In addition, the relatively new field of epigenetics has demonstrated the key role that the environment can play on imprinting plant response to abiotic stress (Kumar, 2018).

Future studies will need to better understand the cross-talk that occurs between different plant developmental processes and how it can be manipulated in a prescribed manner. A key question will be whether processes that determine cold hardiness can be separated from processes that restrict growth. Can *CBF* genes be regulated in a manner that removes their negative impact on growth and reproductive output? What are the genetic mechanisms that can be used to tease these processes apart? Which *CBF* gene or gene variant present in more frost tolerant species is best targeted for manipulation and how can epigenetic modifications affecting their activity best be harnessed?

Although, not as glamorous, a comprehensive understanding of the underlying biophysical mechanisms responsible for freeze avoidance, especially in woody plants, is still lacking. Deep supercooling of xylem parenchyma (Wisniewski, 1995; Fujikawa et al., 2009) and floral buds (Kuprian et al., 2016, 2017) is an integral aspect of the cold hardiness of many temperate tree species, especially fruit trees, however, few advances have been made on this topic over the past 30 years. What new technologies that can be applied to better understand how, when, and where ice is initiated in plants, how it is propagated, and how the size and shape of ice crystals are regulated. Genetic studies of the inheritance of avoidance traits, such as supercooling, have yet to be conducted, but would provide very useful information. An integrated approach that takes into account the complexity of traits that contribute to plant cold hardiness will be needed to achieve advances that can be translated into practical solutions that address the challenges of a rapidly changing climate.

## Author Contributions

MW was responsible for the general overview of the opinions stated in the manuscript and any faults or shortcoming in logic directly fall on him. AN contributed the overview of CBF regulation, and RA the information on the importance of deacclimation in a changing climate. All authors reviewed and agreed with the final version of the submitted manuscript.

## Conflict of Interest Statement

The authors declare that the research was conducted in the absence of any commercial or financial relationships that could be construed as a potential conflict of interest.
